# Arid5a Regulation and the Roles of Arid5a in the Inflammatory Response and Disease

**DOI:** 10.3389/fimmu.2019.02790

**Published:** 2019-12-05

**Authors:** Kishan Kumar Nyati, Riddhi Girdhar Agarwal, Praveen Sharma, Tadamitsu Kishimoto

**Affiliations:** ^1^Laboratory of Immune Regulation, Immunology Frontier Research Center, Osaka University, Osaka, Japan; ^2^Department of Biochemistry, All India Institute of Medical Sciences, Jodhpur, India

**Keywords:** mRNA stability, posttranscriptional regulation, Arid5a, TLR4, inflammation, immune regulation

## Abstract

Abnormal gene expression patterns underlie many diseases that represent major public health concerns and robust therapeutic challenges. Posttranscriptional gene regulation by RNA-binding proteins (RBPs) is well-recognized, and the biological functions of RBPs have been implicated in many diseases, such as autoimmune diseases, inflammatory diseases, and cancer. However, a complete understanding of the regulation mediated by several RBPs is lacking. During the past few years, a novel role of AT-rich interactive domain-containing protein 5a (Arid5a) as an RBP is being investigated in the field of immunology owing to binding of Arid5a protein to the 3′ untranslated region (UTR) of *Il-6* mRNA. Indeed, Arid5a is a dynamic molecule because upon inflammation, it translocates to the cytoplasm and stabilizes a variety of inflammatory mRNA transcripts, including *Il-6, Stat3, Ox40, T-bet*, and IL-17-induced targets, and contributes to the inflammatory response and a variety of diseases. TLR4-activated NF-κB and MAPK pathways are involved in regulating Arid5a expression from synthesis to degradation, and even a slight alteration in these pathways can lead to intense production of inflammatory molecules, such as IL-6, which may further contribute to the development of inflammatory diseases such as sepsis and experimental autoimmune encephalomyelitis. This review highlights the regulation of the Arid5a expression and function. Additionally, recent findings on Arid5a are discussed to further our understanding of this molecule, which may be a promising therapeutic target for inflammatory diseases.

## Introduction

Toll-like receptors (TLRs) comprise a subgroup of pattern-recognition receptors (PRRs) that detect conserved molecular structures of pathogens that are recognized as danger signals by certain immune cells (1). TLR4 is a well-studied PRR. For example, the classic inflammatory stimulation by lipopolysaccharide (LPS) is mediated via TLR4, which can simultaneously activate two distinct innate immune response signaling pathways: one pathway activated by the adaptors toll/interleukin-1-receptor (TIR)-domain containing adaptor protein (TIRAP) and myeloid differentiation primary response 88 (MyD88) and another activated by the adaptors TIR-domain containing adaptor protein inducing interferon-β (TRIF) and TRIF-related adaptor molecule (TRAM) (2). These signaling pathways in turn activate several transcription factors (TFs), including nuclear factor kappa light-chain-enhancer of activated B cells (NF-κB), signal transducer and activator of transcription (STATs), and activator protein-1 (AP-1), that are involved in the induction of proinflammatory cytokines, such as tumor necrosis factor (TNF)-α, interlukin (IL)-6, and IL-1β (3). However, an unsolicited or exaggerated immune response can result in the overproduction and dysregulated release of these cytokines, possibly evoking “cytokine release syndrome” (4). This syndrome is associated with the progression of many infectious and non-infectious diseases, including viral infections such as influenza as well as systemic inflammatory response syndrome, septic shock, tissue injury, multiple sclerosis, and pancreatitis (5). One of the possible explanations for cytokine overproduction and fatal systemic inflammation is deregulation of the posttranscriptional control of cytokine gene expression or, in particular, an imbalance in cytokine mRNA stability regulated by RNA-binding proteins (RBPs) (6). As RBPs play key roles in posttranscriptional events, they are therefore important regulators of the immune response, controlling gene expression by several mechanisms, such as splicing, localization, translation, polyadenylation, and target mRNA decay (7). RBPs regulate the mRNA transcripts of cytokines through both the 5′ and 3′ untranslated regions (UTRs). The 5′UTR dictates the initiation of mRNA translation, and AU-rich elements (AREs) and stem-loop structures in the 3′UTR determine mRNA stability (8, 9). AT-rich interactive domain-containing protein 5a (Arid5a) was identified as an RBP during an analysis of the mechanism of chlorpromazine (CPZ)-induced inhibition of IL-6 (10). Arid5a is a member of the Arid family of proteins, which contain a helix-turn-helix Arid domain and have the ability to bind to nucleic acids (11, 12). Arid5a resides in the nucleus under normal conditions; however, in response to inflammation (13), Arid5a is translocated to the cytoplasm where it is involved in stabilization of the mRNAs of inflammatory cytokines such as IL-6. High levels of inflammatory cytokines augment inflammatory responses and contribute to various autoimmune diseases. Indeed, by stabilizing mRNAs of several inflammatory molecules, Arid5a has been shown to regulate the development of inflammatory and autoimmune diseases (10).

Therefore, control of Arid5a is essential. In this review, we discuss TLR4-mediated regulatory pathways of Arid5a and associations of Arid5a with inflammation and human diseases to further our understanding of this protein, which has the potential to serve as a pioneering target for IL-6-mediated autoimmune and inflammatory diseases.

## Regulation of Arid5a Expression

LPS is a well-known ligand of TLR4 (14–18), and their interaction elicits a downstream signaling cascade that ultimately terminates in the activation of several TFs, including NF-κB and interferon (IFN) regulatory factors (IRFs). These TFs further induce expression of various other inflammatory genes, such as *Il-12, Tnf-*α, and *Irf3*, and initiate appropriate innate and adaptive immune responses (19). Although *Arid5a* expression is induced in the early phase of TLR4 signaling, expression of this gene is rapidly inhibited in the late phase (20). Below, the signaling pathways that control Arid5a expression following LPS induction are discussed.

### Gene Regulation

TLR4 signaling activates inhibitor of kappa B (IκB) kinase (IKK) and p38 mitogen-activated protein kinase (MAPK) and results in the robust production of inflammatory cytokines, and NF-κB is a downstream molecule in IKK signaling. NF-κB family TFs form homo- and heterodimers and include the Rel family members RelB, c-Rel, p65, p50, and p52 (21). Three subunits, c-Rel, p65, and RelB, actively participate in transcriptional regulation, whereas the other two subunits, p50 and p52, predominantly act as non-transactivating DNA-binding subunits (21). As acetylated or phosphorylated p65 exhibits increased transcriptional activity, the function of NF-κB p65 is under the control of posttranslational modification (22).

**Figure 1:**
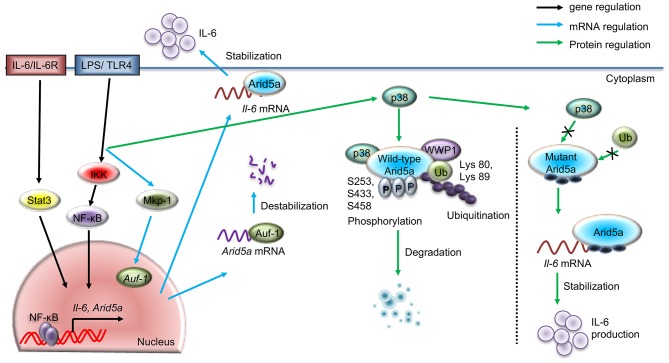
Regulatory pathways for Arid5a under TLR4 signaling. The schematic diagram shows the gene expression of *Arid5a* (black arrow) induced by the TLR4/IKK/NF-κB and IL-6/STAT3 signaling pathways. LPS-bound TLR4 leads to the release of NF-κB from the IKK complex and activates *Arid5a* and other inflammatory genes, such as *Il-6*. Arid5a then stabilizes *Il-6* mRNA (blue arrow), increasing the production of IL-6. Another pathway shows the posttranscriptional regulation of *Arid5a* mRNA (blue arrow), in which TLR4 signaling stimulates MKP-1, which in turn directs AUF-1 to translocate to the cytoplasm. Cytoplasmic AUF-1 binds to AU-rich elements present in the 3′UTR of the *Arid5a* mRNA transcript, which results in destabilization of the *Arid5a* mRNA (blue arrow). The LPS/TLR4 pathway activates p38, leading to phosphorylation of the Arid5a protein at serine residues 253, 433, and 458. This phosphorylation is associated with the ubiquitination of Arid5a at lysines 80 and 89 by WWP1 E3 ligase and subsequent degradation of the protein.

Overexpression of NF-κB (p65/c-Rel) results in an increase in *Arid5a* promoter activity in luciferase assays (20), suggesting the importance of NF-κB for *Arid5a* expression. Furthermore, a mutant of p65 that cannot be acetylated results in reduced *Arid5a* promoter activity, though an unphosphorylatable mutant does not (20). This finding indicates the involvement of p65 acetylation in the transcriptional activation of the *Arid5a* promoter. Collectively, activation of the *Arid5a* gene by NF-κB (p65 and c-Rel), which is mediated by tight binding to the *Arid5a* promoter region and IKK-mediated NF-κB signaling, plays a key role in the regulation of *Arid5a* gene expression (Figure 1).

It has also been shown that *Arid5a* mRNA expression in macrophages and mouse embryonic fibroblasts (MEFs) is enhanced upon IL-6 exposure (10). Furthermore, *Arid5a* mRNA expression is inhibited in *Il-6-*knockdown MEFs activated by LPS (20), though *Arid5a* mRNA expression cannot be induced in *Stat3*-knockdown MEFs, even upon LPS stimulation. These findings suggest that Stat3 plays an essential role in *Arid5a* expression. Further investigation has revealed that phosphorylated Stat3 binds to the promoter region of *Arid5a*, increasing its expression (20). Taken together, these findings indicate that the IL-6 positive feedback loop promotes *Arid5a* expression through activation of Stat3 mediated by TLR signaling (Figure 1).

### mRNA Regulation

As discussed above, TLR4 signaling activates the Arid5a gene. A previous study examined posttranscriptional mechanisms of Arid5a mRNA regulation influenced by TLR4 signaling. An RBP assay coupled with mass spectrometry indicated that ARE/poly(U)-binding/degradation factor 1 (AUF-1), an RBP, is a prominent candidate that binds to the *Arid5a* 3′UTR of the *Arid5a* mRNA transcript in LPS-activated peritoneal macrophages. *Arid5a* mRNA expression has been found to be inhibited in LPS-treated *AUF-1*-knockdown MEFs. AUF-1 is known to bind to AREs present in the 3′UTR and to have the ability to destabilize ARE-containing mRNAs (23). Furthermore, overexpression of AUF-1 decreases the luciferase activity of the *Arid5a* 3′UTR, which contains AREs, suggesting that AUF-1 binds to AREs in the *Arid5a* mRNA. In an mRNA stability assay, *Arid5a* mRNA had a prolonged half-life in actinomycin D-treated *AUF-1*-knockdown MEFs. In addition, slower electrophoretic mobility of the AUF-1-*Arid5a* mRNA complex was found compared to *Arid5a* mRNA alone. It is likely that AUF-1 is able to bind physically to the AREs in the *Arid5a* mRNA 3′UTR and destabilize it (Figure 1).

Evidence shows that MKP-1, a MAPK phosphatase, promotes the nuclear export of AUF-1 to the cytoplasm in response to LPS stimulation; AUF-1 in turn binds to various cytokine mRNAs, such as those encoding TNF-α, IL-10, and IL-6, to regulate mRNA stability (24). Furthermore, *MKP-1*-knockdown MEFs displayed delayed degradation of *Arid5a* mRNA, enhancing *Arid5a* expression. Thus, *MKP-1*-knockdown suppresses cytoplasmic translocation of the AUF-1 protein and increases the stability of the *Arid5a* mRNA. Moreover, LPS stimulation causes MKP-1 to enhance AUF-1 migration from the nucleus to the cytoplasm, where it binds to AREs in the 3′UTR of *Arid5a* mRNA to destabilize it (Figure 1). Nonetheless, the detailed mechanisms remain unknown, particularly with regard to how AUF-1 destabilizes *Arid5a* mRNA, which, for example, may occur via a deadenylation-dependent decay pathway for mRNA degradation.

### Protein Regulation

Phosphorylation and ubiquitination regulate Arid5a posttranslationally, controlling levels of the protein. The Arid5a protein has been found to gradually decrease and nearly vanish in the late phase of LPS stimulation (10, 20). In addition, mass spectrometry analysis revealed that the Arid5a protein is phosphorylated at serine-253,−433, and−458 after TLR4 activation (20), and the Arid5a protein remains unphosphorylated upon mutation of these serine residues, even under LPS stimulation.

Phosphorylation is sometimes accompanied by ubiquitination, which generally results in protein degradation. For example, inflammatory stimulus-activated Regnase-1 is phosphorylated by the IKKα-IKKβ complex and subsequently undergoes ubiquitination and proteasome-mediated degradation (25). In addition to IKKα/β, p38 MAPK regulates the functions of RBPs, such as phosphorylation-induced degradation of substrates and inflammatory cytokine expression (26, 27). For example, p38 MAPK phosphorylates tristetraprolin and facilitates the degradation of *TNF-*α mRNA (28); p38 MAPK signaling also stabilizes the mRNA of the cell cycle regulatory protein p21^Cip1^ by phosphorylating the RBP human antigen R (HuR) (29). Additionally, p300, a co-TF associated with chromatin remodeling, is phosphorylated by p38 MAPK at Ser-1834, which ultimately promotes p300 degradation (30, 31). Interestingly, coexpression of p38 with ubiquitin and Arid5a results in ubiquitination of the Arid5a protein at lysine-80 and−89, unveiling a key role for p38 in the ubiquitination of Arid5a (20). Furthermore, a phosphorylated mutant of Arid5a is not ubiquitinated in the presence of p38, which further suggests that phosphorylation of Arid5a renders susceptible to ubiquitination (Figure 1).

E3 ubiquitin ligases are normally associated with the final step of ubiquitination. A previous study reported a lack of Arid5a ubiquitination in WW domain containing E3 ubiquitin protein ligase 1 (*WWP1*)-knockdown MEFs, indicating that WWP1 is an E3 ligase targeting Arid5a (20). Mechanistically, Arid5a, WWP1, and p38 interact to form a complex, inducing the ubiquitination of Arid5a in response to TLR4 signaling, which in turn decreases *Il-6* mRNA stability. However, mutation of specific phosphorylation sites in Arid5a inhibits its degradation, as p38 and WWP1 fail to interact with these mutated Arid5a derivatives, which ultimately results in overproduction of IL-6 (20) (Figure 1). Because the level of IL-6 is enhanced by Arid5a-mediated *Il-6* mRNA stabilization (Figure 1) (10), WWP1-deficient cells exhibit significant augmentation in IL-6 expression in response to LPS exposure (20). Thus, WWP1-mediated ubiquitination of Arid5a affects *Il-6* stability through the LPS pathway.

## Regulation of Arid5a Function

The function of Arid5a varies depending on its location. Along with other nuclear proteins in resting cells, Arid5a is localized mainly in the nucleus where it regulates the functions of TFs (13). As mentioned above, Arid5a translocates to the cytoplasm under inflammatory conditions (13, 20), where it functions as an mRNA stabilizer (13). Arid5a is abundantly expressed in cardiovascular tissues and acts as an estrogen receptor (ER)-interacting repressor of gene expression in the nucleus (32) by binding to the N- and C-termini of ER-alpha. Arid5a is also expressed in cartilage and induces chondrocyte differentiation as a transcriptional partner of SRY-box transcription factor 9 (Sox9), which regulates chondrocyte differentiation through activation of collagen type 2 alpha 1 (Col2a1), a chondrocyte-specific gene (33). Specifically, Arid5a enhances transcription of the *Col2a1* gene by binding directly to its promoter, which stimulates acetylation of histone 3 proteins located in the region to regulate chondrocyte differentiation in collaboration with Sox9. Furthermore, Arid5a inhibits transcription of the major immediate early (M-IE) gene of human cytomegalovirus (HCMV) by binding to multiple sites in the modulator region, which is located upstream (33).

**Figure 2:**
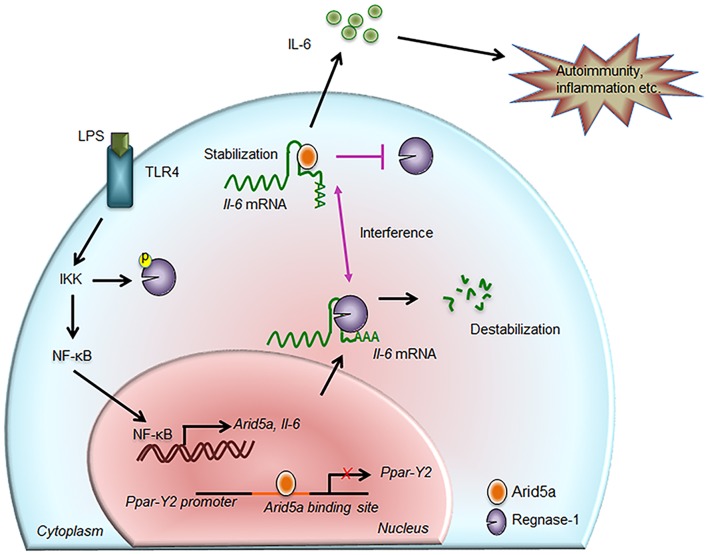
Contribution of Arid5a in IL-6 production and disease. The innate immune response is generated by pathogen-associated molecular patterns, which are recognized by pathogen recognition receptors and lead to expression of proinflammatory mediators. For example, toll-like receptor 4 recognizes lipopolysaccharide and activates the IKK/NF-κB signaling pathway. The IKK complex phosphorylates Regnase-1 and also promotes transcription of *Il-6* and *Arid5a*. Regnase-1 binds to the *Il-6* mRNA and degrades it. In the nucleus, Arid5a binds to genes encoding transcription factors, such as Ppar-γ2, to inhibit their expression. Furthermore, Arid5a is shuttled to the cytoplasm under inflammatory conditions where it interferes with the destabilizing effect of Regnase-1. Arid5a is involved in *Il-6* mRNA stabilization, and the resulting increase in IL-6 production is associated with inflammation and autoimmune diseases.

**Figure 3:**
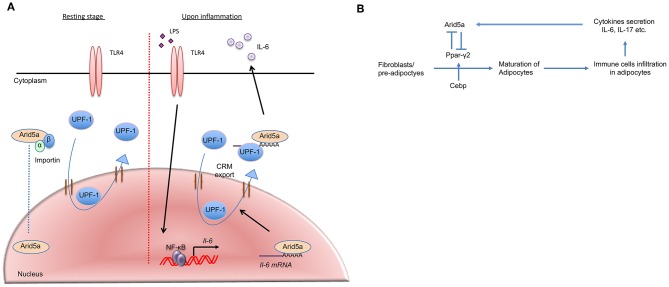
Arid5a translocation and association with adipogenesis and obesity. **(A)** In the resting stage, Arid5a usually resides in the nucleus. Arid5a is imported via an importin-α/β1 pathway. UPF1 shuttles between the nucleus and the cytoplasm. During inflammation, toll-like receptor 4 signaling is activated by lipopolysaccharide, and *Il-6* expression is induced by NF-κB. Arid5a interacts with the *Il-6* mRNA, and Arid5a is subsequently exported to the cytoplasm via the CRM1 pathway with UPF1. **(B)** Arid5a and Ppar-γ2 in adipogenic homeostasis. Cebp activates *Ppar-*γ*2*, and both are involved in adipogenesis. Immune cells, such as macrophages and T cells, infiltrate adipose tissues and provide a framework to regulate energy homeostasis. In adipose tissue, these cells secrete cytokines, such as IL-6 and IL-17, which induce Arid5a. Arid5a further represses *Ppar-*γ*2* transcription in fibroblasts, thereby inhibiting adipocyte development and inhibiting adipogenesis and obesity.

As stated above, Arid5a regulates mRNA stability through binding to AREs and stem-loop structures in the 3′UTRs of target mRNAs, similar to other RBPs. Due to its ability to bind to these regions, the novel function of Arid5a in mRNA stability was first identified when it was found to bind to the *Il-6* 3′UTR in the cytoplasm, promoting the production of IL-6 *in vivo* and inducing experimental autoimmune encephalomyelitis (EAE) (10). The binding of Arid5a to the *Il-6* 3′UTR also protected *Il-6* mRNA transcripts from degradation by the RNA-destabilizing protein Regnase-1 (also known as Zc3h12a) (34), as Regnase-1 and Arid5a interact with the same stem-loop region in the 3′UTR of the *Il-6* mRNA transcript (10) (Figure 2). Moreover, Regnase-1, an endonuclease, was shown to destabilize numerous mRNAs including *Il-6* by binding to a conserved stem-loop structure in the 3′UTR (25). The IKK complex controls *Il-6* mRNA stabilization via Regnase-1 phosphorylation in response to TLR or IL-1 stimulation (25). Regnase-1 acts as an RNase to *Il-6* 3′UTR (1–142 and 58–173), and interestingly, binding of Arid5a to the same site (122–197) prevents its degradation from Regnase-1 (10). Therefore, Arid5a interferes with the destabilization effect of Regnase-1 and contributes to the overproduction of IL-6 *in vivo* (10). Moreover, the predominance of Arid5a over Reganse-1 prolongs *Il-6* mRNA half-life; therefore, Arid5a contributes to the development of autoimmune inflammatory diseases (10). Additionally, Regnase-1 requires up-frameshift protein-1 (UPF-1, an RNA helicase and ATPase) to mediate mRNA decay. UPF-1 is an exportin that mediates the transport of many proteins, including tumor suppressors, growth regulators/proinflammatory proteins, and anti-apoptotic proteins (35). UPF-1 associates with the Arid domain in the N-terminal region (amino acids 1–150) of Arid5a to promote export of the protein to the cytoplasm via the protein chromosomal region maintenance 1 (CRM1) (13) (Figure 3A). Therefore, Arid5a can increase the half-life of *Il-6* mRNA and eventually sustain IL-6 overproduction. Overall, Arid5a plays important roles in promoting inflammation and autoimmune diseases (Figure 2).

Mechanistically, the N-terminal region of Arid5a, which includes the Arid domain, associates with UPF1, which is essential for nonsense-mediated mRNA decay (NMD) and Regnase-1-mediated mRNA decay (36). UPF-1 migrates from the nucleus to the cytoplasm via CRM-1, playing essential roles in both compartments (37, 38). NF-κB/IκBα complexes translocate between the cytoplasm and nucleus via classical nuclear localization sequences (cNLS)-dependent nuclear import and CRM1-dependent nuclear export. In addition, the CRM1 inhibitor leptomycin B (LMB) causes NF-κB/IκBα complexes to be trapped inside the nucleus (39), leading to NF-κB inactivation. Conceivably, NF-κB inactivation may result in inhibition of Arid5a nuclear export. Because NF-κB is activated in response to LPS stimulation, as confirmed by monitoring *Il-6* mRNA production in the nucleus, LMB is added after LPS stimulation. LMB inhibits the nuclear export of Arid5a, which interacts with *Il-6* mRNA and UPF1 after LPS stimulation (13). Thus, it has been suggested that Arid5a translocation from the nucleus to the cytoplasm depends on the UPF1/*Il-6* mRNA/CRM1-mediated pathway under stimulatory conditions (Figure 3A). Collectively, the results of these studies suggest that the functions of Arid5a differ depending on its localization: cytoplasmic Arid5a promotes mRNA stabilization by suppressing the function of UPF1, whereas nuclear Arid5a is associated with the functions of TFs.

Similar to *Il-6*, Arid5a binds to the stem structure of the *Stat3* 3′UTR (1738-1765) via R128 to stabilize the transcript. Stat3 plays a key role in regulating the fate of naive CD4^+^ T cells (23), and in Arid5a-deficient mice, the rate at which naive CD4^+^ T cells differentiate into helper T (Th)17 cells is reduced due to the lower level of IL-17-producing T cells and hence the relatively low expression of *IL-17A*. Accordingly, the level of Stat3 in *Arid5a*^−/−^ T cells critically contributes to impairment of Th17 cell differentiation. Furthermore, *in vitro* overexpression of Stat3 in *Arid5a*^−/−^ T cells reportedly significantly rescues Th17 cell populations. Interaction of Arid5a with the *Stat3* mRNA transcript also prevents Regnase-1 from binding to the stem-loop region of the 3′UTR (40). Additionally, Arid5a functions in the stabilization of *Ox40* mRNA (41), as it binds to the stem-loop of this transcript, impairing the destabilizing effects of Regnase-1 on *Ox40* mRNA. Another study reported that depleting Stat3 under Th17 conditions reduced the mRNA levels of *Ox40* (41). Hence, Stat3 has a direct regulatory function in *Ox40* mRNA expression. Under the influence of Arid5a, the activity of Stat3 and differentiation of Th17 cells are unrepressed (41); thus, balance between the functions of Arid5a and Regnase-1 is essential for proper regulation of Ox40 and Stat3 in CD4^+^ T cells under Th17 cell conditions.

While investigating the role of Arid5a in LPS-induced systemic inflammation, our group previously identified T-box expressed in T cells (T-bet) as another target of Arid5a (42). The study revealed a lack of normal expression of IFN-γ in *Arid5a*-deficient T cells under Th1 cell conditions, with a decrease in the expression of *T-bet* mRNA. T-bet is a TF that plays a major role in regulating IFN-γ production in T cells (43, 44). Mechanistically, Arid5a stabilizes *T-bet* mRNA by binding to the conserved stem-loop structure of its 3′UTR (42), enhancing the production of IFN-γ. IL-6 and IFN-γ are among the major proinflammatory cytokines with augmented expression due to “cytokine release syndrome,” which develops during the pathogenesis of systemic inflammation induced by LPS (4). Thus, Arid5a can bind to the mRNAs of several TFs and cytokines that have AREs and stem-loop structures in their 3′UTR, exacerbating inflammation and diseases by stabilizing these mRNAs.

## Role of Arid5a in Diseases

In activated macrophages and T cells, Arid5a elicits cytokine overproduction by controlling the mRNA stability of proinflammatory mediators such as IL-6, Stat3, T-bet, and Ox40; Arid5a is also expected to be involved in cytokine-mediated diseases. The association of Arid5a with several life-threatening diseases is discussed in the following section.

### LPS-Induced Systemic Inflammation

Zaman et al. (42) showed that Arid5a-deficient T cells were defective in expressing IFN-γ under Th1 cell conditions, with inhibition of *T-bet* mRNA expression. Mice deficient in *Arid5a* expression are reported to be resistant to LPS-induced endotoxic shock, with inhibited expression of proinflammatory cytokines such as TNF-α, IFN-γ, and IL-6 (42). Furthermore, compared to endogenous expression of *Arid5a* in mice, depletion of Arid5a in mice was reported to aid in the recovery of the lungs, spleen, and liver during endotoxic shock (42). Arid5a-deficient mice are also resistant to *Propionibacterium acnes*-primed LPS injection, which is considered to be a T cell-mediated IFN-γ-dependent mouse model of endotoxic shock (45).

### Bleomycin-Induced Lung Injury

A recent study reported that *Arid5a*-deficient mice were resistant to bleomycin-induced lung injury through inhibition of reactive oxygen species (ROS) and exhibited minimal inflammation or edema in lung tissues (46). Moreover, abnormal production of ROS reduced oxidized 1-palmitoyl-2-arachidonoyl-sn-glycero-3-phosphocholine (OxPAPC) production, which, in turn, decreased IL-6 levels, presumably due to the loss of posttranscriptional regulation by Arid5a (46). Thus, regulation by Arid5a may improve current treatment regimens for inflammatory diseases; however, the complete molecular mechanism underlying how Arid5a regulates ROS production remains elusive.

### EAE

*Arid5a*-deficient mice show reduced IL-6 levels due to the loss of *Il-6* mRNA stabilization and display a relatively high resistance to developing EAE (10). Interestingly, in a previous study, CD4^+^ T cells that produced IL-17 were found to be less abundant in *Arid5a*-deficient mice than in wild-type mice. *Stat3* expression decreases in an IL-6-dependent manner in activated *Arid5a*-deficient CD4^+^ T cells (40). *Ox40* expression has also been associated with inflammatory CD4^+^ T cells in EAE (47, 48), promoting IL-17 expression (49, 50). Furthermore, under Th17 conditions, low levels of Ox40 do not lead to enhanced levels of IL-17 in T cells obtained from *Arid5a*-knockout mice. Adoptive transfer of *Arid5a*-deficient encephalitogenic CD4^+^ T cells ameliorates EAE due to the influence of the Arid5a/Ox40 axis on IL-17 production in CD4^+^ T cells and EAE development (41). Notably, Arid5a stabilizes *Stat3* and *Ox40* mRNAs by recognizing the stem-loop structure that Regnase-1 binds to; therefore, Arid5a interferes with the mRNA decay function of Regnase-1 in T cells. In CD4^+^ T cells, the mRNA expression and activation of *Stat3* and *Ox40* are remarkably regulated by a balance between Arid5a and Regnase-1 during IL-6 signaling, which might be involved in controlling Th17 cell differentiation.

### Adipogenesis and Obesity

A recent study suggested that cytokines (including IL-6 secreted by immune cells and mature adipocytes) play roles in limiting adipogenesis and obesity (51) and that IL-6 inhibits the differentiation of adipocytes. However, IL-6 does not inhibit this differentiation under Arid5a-deficient conditions, which indicates that IL-6 utilizes Arid5a to inhibit adipogenesis (51). Arid5a is induced by the cytokines IL-6 and IL-17, and because IL-6 is a strong inducer of Arid5a, it is likely that IL-6-deficient mice have insufficient activation of Arid5a, which results in unrestricted adipogenesis and obesity. Therefore, by activating Arid5a, IL-6 is involved in the inhibition of adipogenesis and obesity. Furthermore, following the induction of Arid5a by cytokines, Arid5a can even limit the differentiation of new fibroblasts into adipocytes by inhibiting expression of peroxisome proliferator-activated receptor gamma (Ppar-γ), which mediates lipid uptake and adipogenesis in adipocytes (51); thus, Arid5a maintains the homeostasis of adipose tissue (Figure 3B). As increased Ppar-γ activity is also associated with weight gain in both humans and mice, Ppar-γ promotes obesity. Despite this effect, Ppar-γ has shown anti-inflammatory properties in sepsis, EAE, and bleomycin-induced lung injury (52–54). Interestingly, our previous findings revealed Arid5a-deficient mice to be more resistant to these diseases than wild-type mice (10, 42, 46), highlighting a connection between Ppar-γ and Arid5a. Our group has shown that Arid5a represses Ppar-γ2 transcriptional activity by binding to the AATATT motif in the Ppar-γ2 promoter (Figure 2); this motif is adjacent to the CCAAT sequence that is the binding site of CCAAT-enhancer-binding proteins (CEBP), which is also involved in adipogenesis. Binding of CEBP activates Ppar-γ2 during adipogenesis; in contrast, Arid5a binding to Ppar-γ2 inhibits CEBP binding to the Ppar-γ2 promoter, thus preventing activation of Ppar-γ2 transcription and ultimately restricting the process of adipogenesis. Overall, the coordinated activity of Ppar-γ2 and Arid5a maintains metabolic homeostasis in adipose tissue (Figure 3B).

## Arid5a, a Plausible Therapeutic Target

To efficiently eliminate invading pathogens, activated immune cells produce proinflammatory cytokines, such as TNF-α and IL-1β; however, when immune responses become deregulated, aberrant expression of cytokines, such as IL-6, is induced during inflammation and other conditions, including sepsis and influenza (55, 56). It well-known that overproduction of IL-6 can be life threatening. To confirm findings related to the overproduction of IL-6, it has been reported that IL-6 blockade may be efficacious in preventing the worsening and progression of IL-6-mediated diseases (57). However, in a study in which IL-6 was completely eliminated in murine models, no improvement in the mortality rate of sepsis was reported (58); this was due to the fact that IL-6 serves as an important cytokine in transmitting defense signals from pathogens and tissue damage to stimulate immune responses for host defense (59). Thus, complete elimination of IL-6 in IL-6-induced inflammatory diseases can be fatal, and an alternative strategy to control but not completely eliminate the production of IL-6 is required. Arid5a, which stabilizes *Il-6* mRNA and accounts for the persistent and excessive overexpression of this mRNA, may constitute a potential target for limiting IL-6 levels. Regardless, no drugs or antibodies that target endogenous proteins have been developed to date. Recently, cytosolic antibodies linked to an Fc receptor, such as the E3-ubiquitin ligase tripartite motif containing protein (TRIM)21, have been shown to degrade the corresponding endogenous target proteins through the ubiquitin-proteasome system. This finding has revealed the possibility of using a pathway similar to the TRIM21-elicited ubiquitin-proteasome system to target and degrade endogenous proteins such as Arid5a with anti-Arid5a antibodies. These antibodies can plausibly be conjugated with peptides that have the ability to penetrate cells without interacting with the cell membrane (60, 61) and therefore have the potential to be considered for therapeutic regimes to balance the action of IL-6 and control the intensity of inflammation.

Additionally, various other methods of targeting Arid5a or IL-6 may be developed. Overexpression of IL-6 promotes *Arid5a* expression through Stat3 activation (10), and Arid5a stabilizes *Il-6* and *Stat3* mRNAs after stimulation by IL-6, forming a feedback loop. This loop contributes to the pathogenesis of several inflammatory autoimmune diseases (10, 40) and can also be disrupted by inhibiting one or more of its participants. In addition, Arid5a-mediated stabilization of *Stat3* mRNA has been found to be involved in the differentiation of Th17 cells (40), which are associated with the development of EAE in mice (10). Therefore, because Stat3 activation results in overexpression of Arid5a and IL-6, expression of both *Il-6* and *Arid5a* can be suppressed by using Stat3 inhibitors such as C188-9, a novel small molecule that was recently identified with the help of computational drug design (62). The effect of C188-9 has been studied in tumor growth and inflammation, and significant reductions in both have been observed (62). It is likely that similar types of inhibitors may be efficacious against inflammatory diseases where IL-6 is overproduced, such as sepsis and EAE.

In addition to regulation via the feedback loop, expression of *Arid5a* and thus IL-6 production may be enhanced by stimuli such as IL-1β, IFN-γ, LPS, and OxPAPC (10, 20, 40, 46). Accordingly, strategies to inhibit expression of these stimuli can aid in indirectly suppressing Arid5a expression and be posed as alternate treatment options for IL-6-dependent inflammatory and autoimmune diseases.

CPZ has been reported to selectively inhibit LPS-induced IL-6 production (63, 64) by specifically decreasing the half-life of *Il-6* mRNA in macrophages (63). As it interacts with clathrin-coated pits, which form in the plasma membrane, CPZ functions as an inhibitor of G-protein coupled receptors (GPCRs) and clathrin-mediated endocytosis (65). This blockade of clathrin-mediated endocytosis results in impairment of the TRIF-dependent pathway, which ultimately causes inhibition of IL-6 (64). The TLR4-stimulated TRIF pathway also induces expression of *Arid5a* in IFN-γ-sensitized human macrophages (66). It is likely that overproduction of IL-6 during the inflammatory response may also result from the TRIF-dependent pathway. Thus, agents blocking the TRIF-dependent pathway, such as CPZ, are potential candidates to suppress the effects of acute inflammation.

It has also been reported that dynamic translocation of Arid5a is necessary for inhibition of Regnase-1-mediated *Il-6* mRNA decay (13). Because Arid5a is an RBP that recognizes *Il-6* through a stem-loop structure and leads to *Il-6* stabilization, abnormal expression of the *Il-6* gene, assisted by Arid5a, may be regulated by stimulation-dependent RBPs. This might inhibit the RNA-stabilizing function of Arid5a and provide new insight into the treatment of diseases that result from a heightened IL-6 response.

## Conclusions and Future Perspectives

TLR4-mediated IKK/NF-κB and IL-6/STAT3 signaling pathways regulate *Arid5a* gene transcription. Upon TLR4 activation, MKP-1 becomes activated and translocates an RBP, AUF-1, from the nucleus to the cytoplasm, where it binds to the AREs present in the 3′UTR of the *Arid5a* mRNA. MKP-1 thereby facilitates *Arid5a* mRNA decay. In the late phase of TLR4 activation, p38 MAPK is reactivated, participating in degradation of the Arid5a protein through phosphorylation-associated ubiquitination in a K48-linked manner. This degradation results in reduced IL-6 levels due to destabilization of the *IL-6* mRNA in the absence of Arid5a. Therefore, to regulate IL-6 production and IL-6-dependent diseases, *Arid5a* expression must be appropriately controlled from synthesis to degradation.

As Arid5a is a dynamic protein that translocates from the nucleus to the cytoplasm under different physiological conditions, it plays location-based roles in cells. Under resting conditions, nuclear Arid5a acts as a cofactor of TFs and contributes to cell proliferation and differentiation, whereas cytoplasmic Arid5a controls the half-lives of mRNAs, including *Il-6* and *Stat3*, in ligand-sensitized cells. These cytokines further activate signaling cascades, producing different cytokines that trigger “cytokine release syndrome” during the pathogenesis of inflammatory diseases, such as sepsis. Thus, inhibiting the function of the RBP Arid5a might also be helpful in treating insuperable diseases, such as EAE and sepsis, that involve overproduction of *Il-6, T-bet, Ox40*, and *Stat3* mRNAs, which are all stabilized by Arid5a. In addition, the report of adipogenesis inhibition via counterregulation of Arid5a and Ppar-γ2 opens a new avenue for developing a therapeutic strategy for metabolic disorders such as obesity. However, extensive research is still required before Arid5a can be accepted for routine clinical treatment.

## Author Contributions

KN conceived, designed, and wrote the manuscript. RA helped in the manuscript preparation and literature search. KN, PS, and TK edited and critically evaluated the manuscript.

### Conflict of Interest

The authors declare that the research was conducted in the absence of any commercial or financial relationships that could be construed as a potential conflict of interest.
